# Infratentorial pathology in frontotemporal dementia: cerebellar grey and white matter alterations in FTD phenotypes

**DOI:** 10.1007/s00415-021-10575-w

**Published:** 2021-05-13

**Authors:** Mary Clare McKenna, Rangariroyashe H. Chipika, Stacey Li Hi Shing, Foteini Christidi, Jasmin Lope, Mark A. Doherty, Jennifer C. Hengeveld, Alice Vajda, Russell L. McLaughlin, Orla Hardiman, Siobhan Hutchinson, Peter Bede

**Affiliations:** 1 Computational Neuroimaging Group, Trinity Biomedical Sciences Institute, Trinity College Dublin, Peter Bede, Room 5.43, Pearse Street, Dublin 2, Ireland; 2 Complex Trait Genomics Laboratory, Smurfit Institute of Genetics, Trinity College Dublin, Dublin 2, Ireland; 3 Department of Neurology, St James’s Hospital, Dublin, Ireland

**Keywords:** Frontotemporal dementia, Cerebellum, PPA, Behaviour, MRI, Cortical thickness

## Abstract

The contribution of cerebellar pathology to cognitive and behavioural manifestations is increasingly recognised, but the cerebellar profiles of FTD phenotypes are relatively poorly characterised. A prospective, single-centre imaging study has been undertaken with a high-resolution structural and diffusion tensor protocol to systematically evaluate cerebellar grey and white matter alterations in behavioural-variant FTD(bvFTD), non-fluent variant primary progressive aphasia(nfvPPA), semantic-variant primary progressive aphasia(svPPA), *C9orf72*-positive ALS-FTD(C9 + ALSFTD) and *C9orf72*-negative ALS-FTD(C9-ALSFTD). Cerebellar cortical thickness and complementary morphometric analyses were carried out to appraise atrophy patterns controlling for demographic variables. White matter integrity was assessed in a study-specific white matter skeleton, evaluating three diffusivity metrics: fractional anisotropy (FA), axial diffusivity (AD) and radial diffusivity (RD). Significant cortical thickness reductions were identified in: lobule VII and crus I in bvFTD; lobule VI VII, crus I and II in nfvPPA; and lobule VII, crus I and II in svPPA; lobule IV, VI, VII and Crus I and II in C9 + ALSFTD. Morphometry revealed volume reductions in lobule V in all groups; in addition to lobule VIII in C9 + ALSFTD; lobule VI, VIII and vermis in C9-ALSFTD; lobule V, VII and vermis in bvFTD; and lobule V, VI, VIII and vermis in nfvPPA. Widespread white matter alterations were demonstrated by significant fractional anisotropy, axial diffusivity and radial diffusivity changes in each FTD phenotype that were more focal in those with C9 + ALSFTD and svPPA. Our findings indicate that FTD subtypes are associated with phenotype-specific cerebellar signatures with the selective involvement of specific lobules instead of global cerebellar atrophy.

## Introduction

The function of the cerebellum continues to be defined, particularly with respect to its physiological role in cognition and behaviour. Clinical observations from acquired cerebellar pathologies have consistently highlighted the posterior predominance of cognitive functioning in the cerebellum [1, 2] and imaging studies have confirmed the specific role of lobules VI, VIIA, VIIB, IX and crus I/II in mediating cognitive processes [3–5]. Posterior cerebellar injuries may manifest in multi-domain cognitive deficits including verbal memory, language, visuospatial, executive function and sequencing abilities; while cognition may be relatively preserved in those with anterior cerebellar insults [6]. Cerebellar pathology may contribute to impairments in social cognition [7], language deficits [8] and pathological crying and laughing [9, 10]. Lesions of the vermis have been linked to emotional dysregulation such as irritability, impulsivity and disinhibition [11]. While the neuropsychological sequelae of acute vascular, neoplastic and inflammatory cerebellar pathologies are widely recognised, cognitive deficits associated with slowly progressive neurodegenerative conditions are less well characterised. There is a striking paucity of imaging data on cerebellar involvement in FTD [12–14] despite ample post-mortem evidence of cerebellar pathology [15]. A recent meta-analysis noted lobule VI, VIIb, VIIIb atrophy in bvFTD, crus I and lobule VI volume loss in svPPA [16]. Genetic FTD subtypes appear to exhibit specific grey matter cerebellar abnormalities [12–14, 17, 18]. The *C9orf72* genotype has been linked to focal crus I and lobule VIIa degeneration, MAPT mutation associated with vermis pathology, and GRN mutation with relatively preserved cerebellar integrity [13]. Interestingly, regional cerebellar atrophy was detected in asymptomatic *C9orf72* mutation carriers [19]. ALS-FTD has been linked to superior (lobules I–VI), crus and vermis degeneration [20]. Other cerebellar regions, such as the cerebellar crura and lobule VI may be involved in all FTD subtypes [16]. This region is often labelled ‘the cognitive cerebellum’ because of its central role in cognitive processing; the extent of atrophy in this area is thought to correlate with cognitive performance across a multitude of domains [14, 16]. Existing studies suggest that cerebellar abnormalities are most widespread in those with ALS-FTD and bvFTD, and may be relatively focal in those with svPPA or nfvPPA [14, 17, 20]. Selective cerebellar atrophy seems to mirror patterns of cerebral cortical pathology [21, 22] and is likely to be defined by cerebello-cerebral connectivity. These observations further support the ‘dysmetria of thought theory’ whereby cerebellar lesions result in individual patterns of cognitive dysfunction dependent on the cortico-cerebellar tracts involved [23]. The majority of cerebellar imaging studies in FTD solely appraise grey matter alterations, white matter degeneration is less well characterised in vivo, and there is a lack of cerebellar functional and metabolic studies. Cerebellar hypometabolism have been reported [16, 24, 25] but the majority of PET studies focus on supratentorial regions.

Post-mortem studies in FTD also disproportionately focus on supratentorial regions. Much of the limited post-mortem data of cerebellar pathology in FTD pertains to a select cohort of those carrying the *C9orf72* mutation. In such cases, TDP-43 negative, ubiquitin and p62-positive neuronal cytoplasmic inclusions were noted in the granular layer of the cerebellar cortex, but these findings are not exclusive to this genotype [15, 26, 27]. Cerebellar atrophy has been described in those carrying the *C9orf72* gene mutation, but not in those carrying the MAPT mutation [26, 28]. A case series of two sisters with a clinical diagnosis of bvFTD and no established genetic mutation, demonstrated abundant abnormal tau deposition in the cerebellum, with a distinctly different morphology from the more common tauopathies [29].

Emerging imaging and post-mortem data lends credence to the body of evidence that cerebellar involvement may contribute to the clinical manifestations of FTD. These observations provide the rationale to characterise cerebellar signatures in FTD phenotypes using a multiparametric grey and white matter imaging protocol. The main objective of this FTD study is to ascertain if focal cerebellar degeneration may be identified in vivo, and establish phenotype-specific and overlapping radiological features.

## Methods

### Participants

A total of 156 participants were included in a prospective imaging study of frontotemporal dementia; 7 patients with behavioural-variant FTD (‘bvFTD’, mean age 60.71 ± 3.3), 12 patients with non-fluent-variant primary progressive aphasia (‘nfvPPA’, mean age 61.5 ± 2.96), 3 patients with semantic-variant primary progressive aphasia (‘svPPA’, mean age 61.66 ± 6.42), 12 ALS-FTD patients carrying *C9orf72* GGGGCC hexanucleotide repeat expansions (‘C9 + ALSFTD’, mean age 58.65 ± 11.22), 12 *C9orf72*-negative ALS-FTD patients repeats (‘C9-ALSFTD’, mean age 59.95 ± 7.67), and 110 healthy controls (‘HC’, mean age 59.21 ± 10.5). All participants provided informed consent in accordance with the Medical Ethics Approval of the research project (Beaumont Hospital, Dublin, Ireland). Exclusion criteria included prior traumatic brain injury, cerebrovascular events, comorbid neoplastic, paraneoplastic, or neuroinflammatory diagnoses. FTD and ALS-FTD was diagnosed based on the Rascovsky criteria [30] and participating ALS patients had ‘probable’ or ‘definite’ ALS according to the revised El Escorial research criteria. Healthy controls were unrelated to patients and had no known family history of neurodegenerative conditions.

### Magnetic resonance imaging

Imaging data were acquired on a 3 T Philips Achieva Magnetic resonance (MR) platform with an 8-channel receive-only head coil. The standardised imaging protocol included a high-resolution T_1_-weighted (T1w) and a 32-direasction diffusion tensor imaging (DTI). T1w was acquired with a 3D Inversion Recovery prepared Spoiled Gradient Recalled echo (IR-SPGR) sequence with the following parameters; field-of-view (FOV) of 256 × 256 × 160 mm, flip angle = 8°, spatial resolution of 1 mm^3^, SENSE factor = 1.5, TR/TE = 8.5/3.9 ms, TI = 1060 ms. DTI data were acquired with a spin-echo echo planar imaging (SE-EPI) pulse sequence using a 32-direction Stejskal–Tanner diffusion encoding scheme, FOV = 245 × 245 × 150 mm, 60 slices with no interslice gap, spatial resolution = 2.5 mm^3^, TR/TE = 7639/59 ms, SENSE factor = 2.5, *b* values = 0, 1100 s/mm^2^, dynamic stabilisation and spectral presaturation with inversion recovery (SPIR) fat suppression.

### Morphometry

First, total intracranial volumes (TIV) were estimated for each subject to be used as a covariate in subsequent region of interest (ROI) morphometric analyses. As described previously [31, 32], TIV estimation was performed by linearly aligning each participant’s skull-stripped brain image to the MNI152 standard, and the inverse of the determinant of the affine registration matrix was calculated and multiplied by the size of the template. FMRIB’s FSL-FLIRT was used for spatial registration and FSL-FAST for tissue-type segmentation. Partial grey matter, white matter, and CSF volumes were added for TIV estimation. Grey matter pathology in the FTD groups was evaluated by ROI morphometry using FMRIB’s FSL suite. Pre-processing steps included skull removal (BET), motion corrections and tissue-type segmentation [33]. Grey-matter partial volume images were aligned to the MNI152 standard space using affine registration. A study-specific grey matter template was created representing each study group to which the grey matter images of each participant were subsequently non-linearly co-registered. Permutation-based non-parametric inference was utilised to contrast each patient group with healthy control implementing the threshold-free cluster enhancement (TFCE) method. Design matrices included group membership, age, sex and TIV [34]. Statistics were restricted to a cerebellar ROI mask defined by label 1 of the MNI structural atlas. Resulting statistical maps were thresholded at *p* < 0.05 and visualised in FSLeyes. The aid the localisation of statistically significant clusters the Diedrichsen probabilistic atlas was used as undelay [35].

### Cortical thickness analyses

To evaluate cerebellar cortical thickness alterations, the cerebellum was segmented using a validated parcellation algorithm. [36–38] A patch-based segmentation algorithm was then applied to obtain cerebellar GM metrics for each lobule, separately for the right and left cerebellar hemispheres [36]. As a quality-control step, anatomical parcellation and tissue-type segmentation was individually verified for each subject. The following labels were used to retrieve regional cortical thickness values: lobules I–V, lobule VI, lobule VIIb, lobules VIII–X, Crus I, and Crus II. To test the effect of group membership on cerebellar cortical thickness in each lobule, Multivariate analyses of covariance (MANCOVAs) were conducted for the right and left cerebellar hemispheres separately, designating lobular cortical thickness as dependent variable, group membership as independent factor and age and gender as covariates. In case of a significant multivariate omnibus test, post hoc comparisons were considered significant at *p* < 0.05, following false-discovery rate (FDR) corrections for multiple comparisons to reduce Type I error.

### White matter analyses

Raw DTI data underwent eddy current corrections and skull removal before a tensor model was fitted to generate maps of fractional anisotropy (FA), axial diffusivity (AD), and radial diffusivity (RD). The tract-based statistics (TBSS) module of FMRIB’s software library was utilised for non-linear registration and skeletonisation of individual DTI images. A mean FA mask was created and each subject’s individual AD, FA and RD images were merged into four-dimentional (4D) AD, FA and RD image files. The input file order matched the group membership variables in the design matrix. Permutation-based non-parametric inference was used for the two-way, voxelwise comparison of diffusivity parameters between each FTD group and controls using design matrix-defined contrasts which included age and gender as covariates. The study-specific white matter skeleton was masked by atlas-defined labels for the entire cerebellum (left and right hemispheres) to restrict analyses to the cerebellum. The threshold-free cluster enhancement (TFCE) method was applied and results considered significant at a *p* < 0.01 TFCE family-wise error (FWE).

### Genetic testing

Pathogenic GGGGCC hexanucleotide repeat expansions in *C9orf72* were screened for with repeat-primed PCR as described previously [39, 40]. Amplified DNA fragments were evaluated with the Applied Biosystems 3130xl Genetic Analyser (Foster City, CA, USA) and visualised using GeneMapper version 4.0. GGGGCC hexanucleotide repeat expansions longer than 30 were considered positive. Participating patients were also screened and tested negative for other mutations associated with ALS and FTD: *SOD1, ALS2, SETX, SPG11, FUS, VAPB, ANG, TARDBP, FIG4, OPTN, ATXN2, VCP, UBQLN2, SIGMAR1, CHMP2B, PFN1, ERBB4, HNRNPA1, MATR3, CHCHD10, UNC13A, DAO, DCTN1, NEFH, PRPH, SQSTM1, TAF15, SPAST, ELP3, LMNB1, SARM1, C21orf2, NEK1, FUS, CHMP2B, GRN, MAPT, PSEN1, PSEN2, TBK1.*

## Results

### Morphometry

**Fig. 1:**
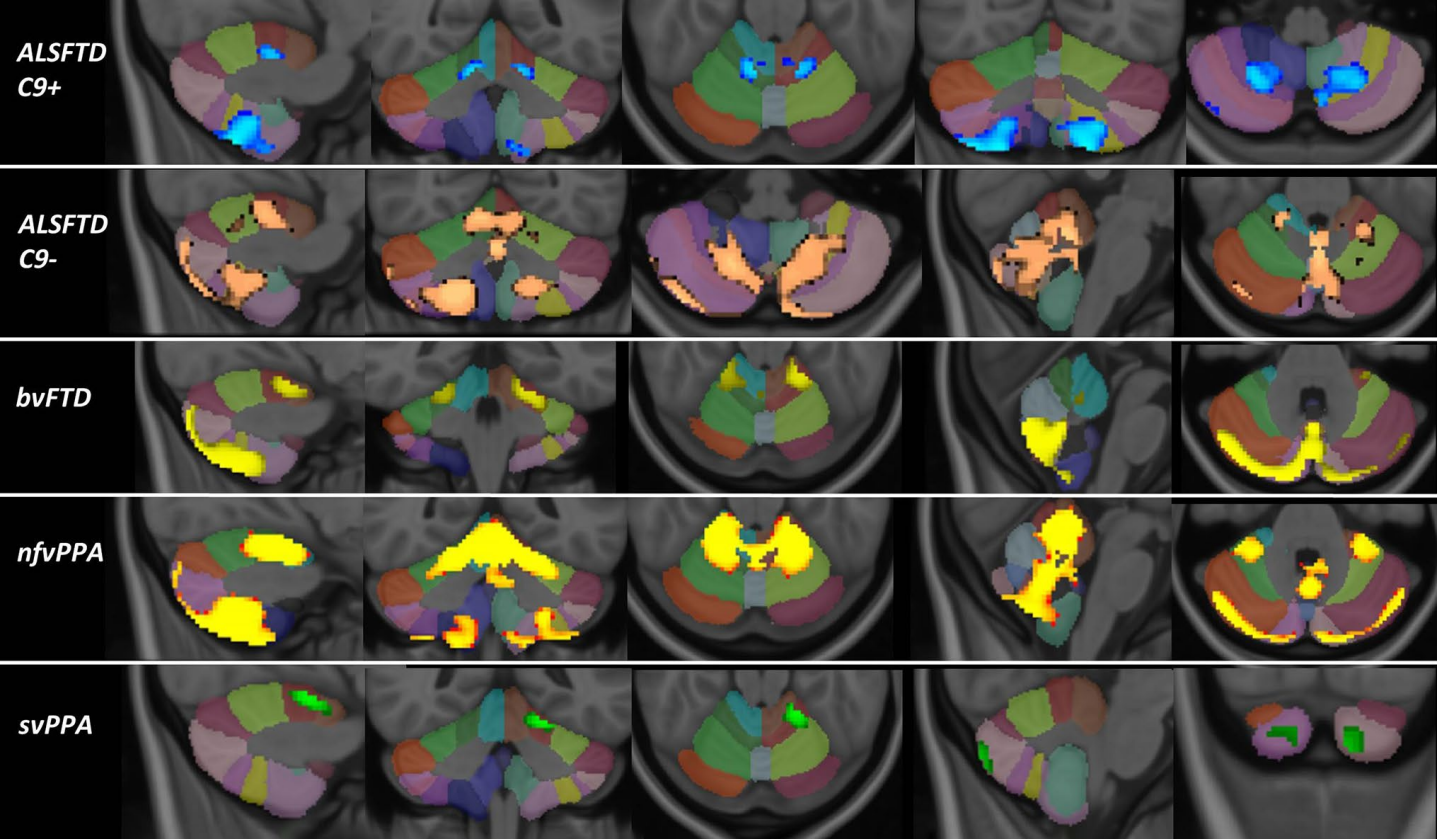
Cerebellar grey matter changes in FTD phenotypes at *p* < 0.05 TFCE corrected for age, gender and TIV. Focal changes in C9 + ALSFTD are indicated in blue, C9-ALSFTD in copper colour, bvFTD in yellow, nfvPPA red-yellow, svPPA in green. The Diedrichsen probabilistic cerebellar atlas is presented as underlay to aid localisation

Region-of-interest morphometry in a study-specific, atlas-defined cerebellar grey matter mask revealed phenotype-specific patterns of atrophy at *p* < 0.05 TFCE (corrected for age, sex and TIV). GGGGCC hexanucleotide repeat carrying ALS-FTD patients exhibited symmetric lobule VIII and lobule V atrophy. *C9orf72*-negative ALS-FTD patients displayed lobule V, VI, VIII and vermis atrophy. Behavioural-variant FTD patients showed vermis, lobule V, lobule VII and symmetric posterior-inferior volume reductions. Non-fluent variant primary progressive aphasia patients exhibited widespread atrophy including lobules V, VI, VIII, and the vermis. Semantic-variant FTD patients displayed volume loss in crus I, Crus II, and lobule V on the left (Fig. 1.)

### Cortical thickness

The evaluation of cortical thickness profiles revealed the preferential involvement of specific cerebellar lobules in FTD phenotypes with the apparent sparing of other cerebellar regions.

Following FDR corrections and statistical adjustments for demographic factors, *C9orf72*-positive ALS-FTD patients exhibited reduced cortical thickness in Lobule IV, VI,VIIb, Crus I and II. Crus II and lobule VI was affected in both cerebellar hemispheres (Table 1). Cortical thinning did not reach statistical significance in *C9orf72*-negative ALS-FTD patients in any of the evaluated cerebellar regions. Patients with behavioural-variant FTD showed cortical thinning in crus I and a trend of thinning post FDR in lobule VII of the right cerebellar hemisphere. Patients with non-fluent variant primary progressive aphasia (nfvPPA) exhibited lobule VI, VIIb, crus I and II. Lobule VI and crus II atrophy was observed in each hemisphere. Patients with semantic-variant FTD (svPPA) showed lobule VIIb, crus I and II degeneration in the right cerebellar hemisphere.

**Table 1:** The cerebellar cortical thickness profile of the ALS-FTD spectrum

Cerebellar lobule		Cortical thickness: estimated marginal mean ± standard error (mm)						Statistics						
		HC	ALS-FTD-C9-	ALS-FTD-C9 +	bvFTD	nvfPPA	svPPA	*F*, *p* value	Univariate effect size	HC vs ALSFTDC9-	HC vs ALSFTDC9 +	HC vs bvFTD	HC vs nfvPPA	HC vs svPPA
Left ^a^	I–II	1.416 ± 0.031	1.678 ± 0.074	1.437 ± 0.074	1.432 ± 0.124	1.604 ± 0.095	1.749 ± 0.190	*F* = 3.045, *p* = 0.012	η^2^*p* = 0.085	**0.015**	0.9	0.94	0.21	0.23
	III	3.213 ± 0.035	3.355 ± 0.083	3.269 ± 0.083	3.058 ± 0.140	3.182 ± 0.107	3.501 ± 0.214	*F* = 1.198, *p* = 0.312	η^2^*p* = 0.035	0.26	0.73	0.47	0.9	0.36
	IV	4.913 ± 0.014	4.930 ± 0.033	4.817 ± 0.033	4.788 ± 0.055	4.891 ± 0.042	4.919 ± 0.084	*F* = 2.462, *p* = 0.035	η^2^*p* = 0.070	0.82	**0.06**^**t**^	0.133	0.82	0.95
	V	4.898 ± 0.015	4.875 ± 0.035	4.803 ± 0.034	4.796 ± 0.058	4.816 ± 0.045	4.873 ± 0.089	*F* = 2.095, *p* = 0.069	η^2^*p* = 0.060	0.73	0.072	0.23	0.23	0.9
	VI	4.978 ± 0.011	4.917 ± 0.026	4.898 ± 0.026	4.907 ± 0.044	4.880 ± 0.034	4.975 ± 0.067	*F* = 3.431, *p* = 0.006	η^2^*p* = 0.095	0.15	**0.05**	0.26	**0.06**^**t**^	0.97
	VIIB	4.608 ± 0.021	4.566 ± 0.049	4.476 ± 0.049	4.470 ± 0.082	4.408 ± 0.063	4.467 ± 0.125	*F* = 3.164, *p* = 0.009	η^2^*p* = 0.088	0.61	0.076	0.26	**0.036**	0.46
	VIIIA	4.649 ± 0.018	4.662 ± 0.041	4.643 ± 0.041	4.502 ± 0.070	4.598 ± 0.053	4.637 ± 0.107	*F* = 1.023, *p* = 0.406	η^2^*p* = 0.030	0.9	0.94	0.168	0.54	0.94
	VIIIB	4.514 ± 0.032	4.671 ± 0.076	4.430 ± 0.076	4.322 ± 0.129	4.711 ± 0.098	4.582 ± 0.196	*F* = 2.290, *p* = 0.048	η^2^*p* = 0.065	0.21	0.49	0.3	0.21	0.9
	IX	3.570 ± 0.043	3.621 ± 0.101	3.398 ± 0.101	3.298 ± 0.170	3.715 ± 0.130	3.504 ± 0.260	*F* = 1.363, *p* = 0.241	η^2^*p* = 0.040	0.82	0.26	0.26	0.47	0.9
	X	2.491 ± 0.042	2.299 ± 0.099	2.468 ± 0.098	2.273 ± 0.0166	2.609 ± 0.127	2.710 ± 0.254	*F* = 1.400, *p* = 0.227	η^2^*p* = 0.041	0.23	0.9	0.38	0.55	0.51
	Crus I	4.575 ± 0.021	4.516 ± 0.049	4.377 ± 0.049	4.445 ± 0.083	4.405 ± 0.063	4.353 ± 0.126	*F* = 4.241, *p* = 0.001	η^2^*p* = 0.114	0.46	**0.003**	0.27	0.072	0.23
	Crus II	4.365 ± 0.026	4.290 ± 0.062	4.097 ± 0.062	4.390 ± 0.104	4.090 ± 0.080	3.983 ± 0.159	*F* = 5.695, *p* < 0.001	η^2^*p* = 0.148	0.46	**0.003**	0.9	**0.015**	0.095
Right^b^	I–II	1.354 ± 0.029	1.636 ± 0.068	1.386 ± 0.068	1.462 ± 0.114	1.569 ± 0.087	1.587 ± 0.174	*F* = 3.878, *p* = 0.002	η^2^*p* = 0.106	**0.001**	0.85	0.56	0.09	0.4
	III	3.092 ± 0.032	3.167 ± 0.076	3.118 ± 0.076	3.163 ± 0.128	3.142 ± 0.098	3.139 ± 0.196	*F* = 0.234, *p* = 0.947	η^2^*p* = 0.007	0.56	0.85	0.8	0.83	0.86
	IV	4.772 ± 0.019	4.755 ± 0.045	4.714 ± 0.045	4.758 ± 0.076	4.819 ± 0.058	4.794 ± 0.116	*F* = 0.475, *p* = 0.795	η^2^*p* = 0.014	0.85	0.45	0.86	0.65	0.86
	V	4.752 ± 0.018	4.679 ± 0.043	4.648 ± 0.043	4.686 ± .072	4.735 ± 0.055	4.703 ± 0.110	*F* = 1.399, *p* = 0.227	η^2^*p* = 0.041	0.26	0.11	0.56	0.85	0.85
	VI	4.928 ± 0.011	4.880 ± 0.026	4.855 ± 0.026	4.883 ± 0.043	4.835 ± 0.033	4.901 ± 0.066	*F* = 2.768, *p* = 0.020	η^2^*p* = 0.078	0.23	**0.054**^**t**^	0.52	**0.054**^**t**^	0.85
	VIIB	4.788 ± 0.017	4.695 ± 0.041	4.608 ± 0.041	4.610 ± 0.069	4.665 ± 0.053	4.399 ± 0.106	*F* = 6.938, *p* < 0.001	η^2^*p* = 0.175	0.14	**0.001**	**0.07**^**t**^	0.11	**0.001**
	VIIIA	4.642 ± 0.017	4.600 ± 0.039	4.571 ± 0.039	4.522 ± 0.066	4.576 ± 0.050	4.448 ± 0.101	*F* = 1.849, *p* = 0.106	η^2^*p* = 0.053	0.52	0.24	0.23	0.44	0.18
	VIIIB	4.573 ± 0.026	4.593 ± 0.061	4.497 ± 0.061	4.454 ± 0.103	4.519 ± 0.079	4.673 ± 0.157	*F* = 0.695, *p* = 0.628	η^2^*p* = 0.021	0.85	0.45	0.45	0.73	0.74
	IX	3.763 ± 0.037	3.70 ± 0.088	3.609 ± 0.088	3.485 ± 0.148	3.720 ± 0.113	3.720 ± 0.226	*F* = 1.148, *p* = 0.337	η^2^*p* = 0.034	0.86	0.26	0.21	0.85	0.86
	X	2.251 ± 0.036	2.105 ± 0.085	2.146 ± 0.085	1.996 ± 0.143	2.228 ± 0.110	2.170 ± 0.219	*F* = 1.127, *p* = 0.348	η^2^*p* = 0.033	0.26	0.45	0.23	0.86	0.85
	Crus I	4.636 ± 0.022	4.572 ± 0.051	4.524 ± 0.051	4.393 ± 0.086	4.429 ± 0.066	4.157 ± 0.131	*F* = 5.628, *p* < 0.001	η^2^*p* = 0.146	0.45	0.15	**0.047**	**0.03**	**0.001**
	Crus II	4.576 ± 0.024	4.499 ± 0.055	4.412 ± 0.055	4.381 ± 0.093	4.364 ± 0.071	4.035 ± 0.142	*F* = 5.637, *p* < 0.001	η^2^*p* = 0.147	0.41	**0.047**	0.15	**0.043**	**0.001**

Estimated marginal means ± S.E. for cortical thickness are adjusted for age and gender

^a^Pillai’s Trace = 0.623; *F* (12,60) = 1.864; *p* < 0.001; η^2^*p* = 0.125;

^b^Pillai’s Trace = 0.575; *F* (12,60) = 1.701; *p* = 0.001; η^2^*p* = 0.115; Bold *p* values are significant at *p* < 0.05, after false-discovery rate correction for multiple comparisons. Partial η^2^ effect size is interpreted as small (η^2^*p* = 0.01), medium (η^2^*p* = 0.06) or large (η^2^*p* = 0.14). ^t^ statistical trend at *p* ≤ 0.07

### White matter alterations

**Fig. 2:**
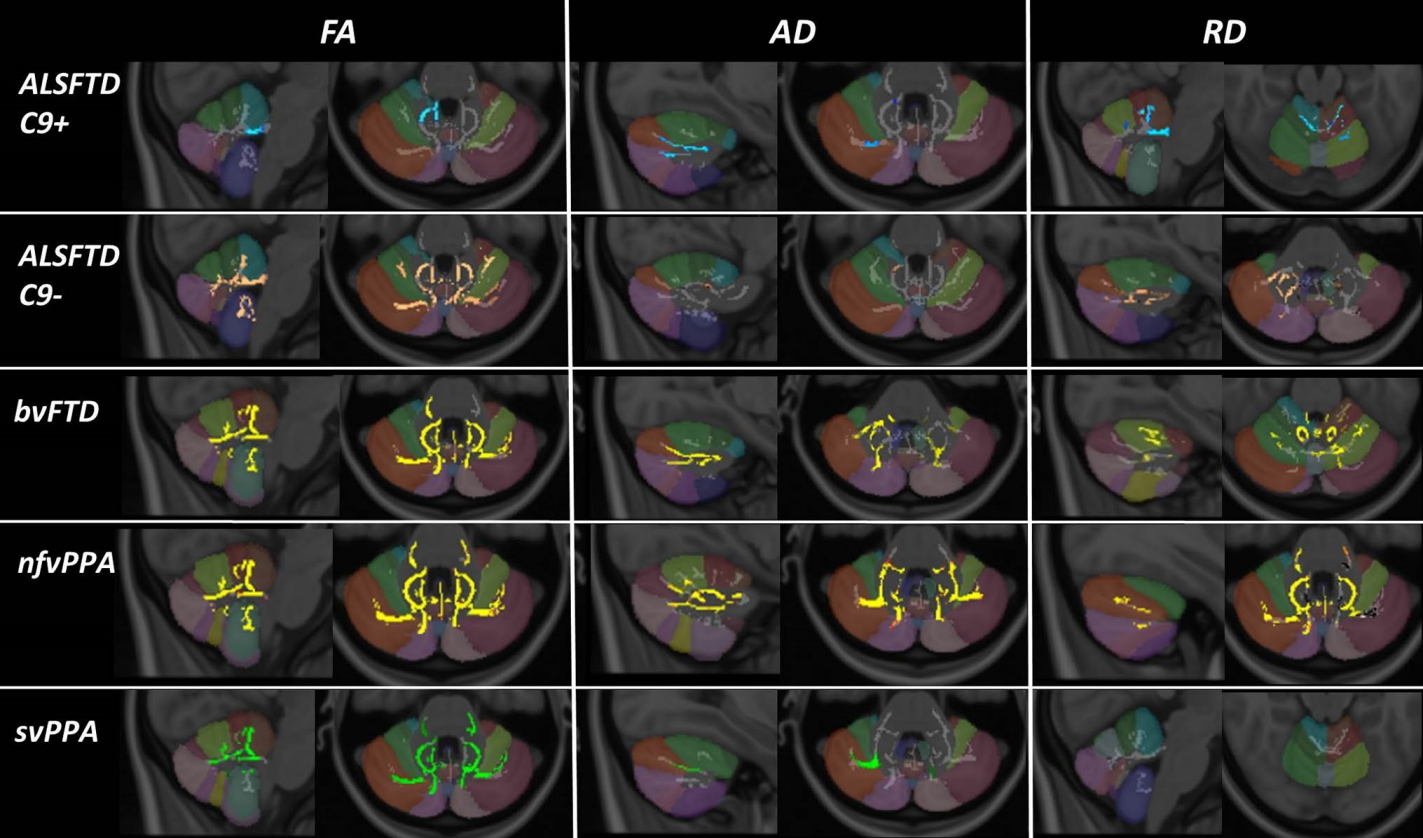
Tract-based white matter changes in FTD phenotypes as identified by FA, AD and RD alterations at *p* < 0.01 TFCE adjusted for age and gender. Changes in C9 + ALSFTD are indicated in blue, C9-ALSFTD in copper colour, bvFTD in yellow, nfvPPA red-yellow, svPPA in green. The Diedrichsen probabilistic cerebellar atlas is presented as underlay to aid localisation

Permutation-based non-parametric statistics confirmed focal diffusivity alterations at *p* < 0.01 TFCE (corrected for age and sex) in a study-specific cerebellar white matter skeleton. Reduced fractional anisotropy, reduced axial diffusivity and increased radial diffusivity were detected in each FTD phenotype with reference to healthy controls. Patterns of white matter vulnerability varied along the ALS-FTD spectrum (Fig. 2). *C9orf72*-positive ALS-FTD patients exhibited reduced FA in the superior cerebellar peduncle, reduced AD in Crus I and II, and increased RD in lobules I–IV as well as in the superior peduncle. *C9orf72*-negative ALS-FTD patients displayed widespread, symmetric, multi-lobular FA reductions, focal AD reduction in the right lobule V, and increased RD in crus I and II in the right cerebellar hemisphere. Patients with behavioural-variant FTD showed FA reductions in nearly the entire cerebellar white matter skeleton, reduced AD in crus I and II, and widespread areas of increased RD in particular in lobule VI. Patients with non-fluent variant primary progressive aphasia (nfvPPA) exhibited multi-lobular FA and AD reductions and similarly widespread RD increases. Patients with semantic-variant FTD (svPPA) showed superior-predominant symmetric FA reductions centred on lobule V, reduced AD in Crus I, and no RD alterations at *p* < 0.01.

### Summary of findings

The integration of findings across multiple imaging modalities revealed the selective involvement of cerebellar regions with relatively distinctive imaging signatures along the ALS-FTD spectrum (Table 2.)

**Table 2:** Summary of focal findings across the five imaging modalities

Study group	Morphometry	FA	AD	RD	Cortical thickness
C9 + ALSFTD	Lobule V, VIII	Superior cerebellar peduncle	Crus I & II	Lobules I–IV superior peduncle	Lobule IV, VI,VII Crus I & II
C9-ALSFTD	Lobule V, VI, VIII, vermis	Widespread multi-lobular	Lobule V	Crus I & II	Nil at *p* < 0.05 post FDR
bvFTD	Lobule V, VII, vermis	widespread multi-lobular	Crus I & II	Widespread multi-lobular	lobule VII, crus I
nfvPPA	Lobule V, VI, VIII, vermis	Widespread multi-lobular	Widespread multi-lobular	Widespread multi-lobular	lobule VI, VII, crus I & II
svPPA	Lobule V, crus I & II	Lobule V, and superior cerebellum	crus I	Nil at *p* < 0.01	Lobule VII, crus I & II

## Discussion

Our study indicates that clinical subtypes of FTD exhibit individual patterns of cerebellar degeneration; these changes are widespread in nfvPPA and bvFTD, but relatively focal in svPPA. Marked cerebellar differences were detected between C9 + ALSFTD and C9-ALSFTD. Our data suggest that certain cerebellar regions, such as lobule V, VI, VIII, vermis, Crus I and II, are more susceptible to degeneration in FTD than other areas. While our findings are in line with previous reports [14, 16], one of the novelty of our study is the detection of lobule V degeneration across the clinical spectrum of FTD. This lobule is part of the anterior cerebellar lobe that primarily mediates sensorimotor functions [4, 41, 42]. However, dichotomising motor and cognitive functions to the anterior and posterior cerebellum may be simplistic; lobule V is also involved in verbal working memory, emotion and rhythm processing [3, 4]. This region has previously been implicated in bvFTD cohorts including those with ALSFTD [12]. We have also demonstrated that the cerebellar vermis is involved in nfvPPA, C9-ALSFTD, and to a greater extent in bvFTD. Vermis degeneration has been previously linked to bvFTD and described in ALSFTD [12, 14, 16, 20]. This region is often referred to as the ‘limbic cerebellum’ because of its role in emotion processing and its connectivity with the limbic and paralimbic regions [3]. Structural abnormalities in this region may manifest in a myriad of irregular social or emotional behaviours, including aggression, irritability and disinhibition [11, 43, 44]. A similar constellation of symptoms may occur in opsoclonus myoclonus syndrome, a post-infectious or paraneoplastic disorder that preferentially involves the cerebellar vermis [45]. These observations are further supported by altered cerebello-cerebral connectivity in bipolar affective disorder [38, 46]. The functional topography of the cerebellum has been gradually elucidated [47] and careful meta-analyses have ascribed specific higher level cognitive functions to distinct cerebellar areas [3, 4]. The affected regions identified in our study within the ‘cognitive cerebellum’ are involved in emotional processing, attention, executive function, working memory, language including expressive language, and social cognition [3, 4, 48]. Functional MRI studies have confirmed the co-activation of posterior cerebellar and prefrontal cortices during cognitive tasks, patterns which are distinctly different from the activation of the anterior cerebellum and sensorimotor cortices during motor tasks [5, 49, 50]. This pattern of connectivity has been replicated in greater detail in post-mortem studies [51, 52].

We predominantly observed symmetric cerebellar degeneration, with the exception right hemisphere dominant cortical thinning in bvFTD and svPPA. The asymmetric cerebellar findings in svPPA may be linked to the similarly lateralised pathology at a supratentorial level and potentially mediated by crossed cerebellar network [3, 4, 14, 53, 54]. It is noteworthy, however, that, exclusively left-sided lobule V, crus I–II volume reductions were noted in svPPA on morphometric analyses. These observations highlight that different imaging modalities capture different aspects of cerebellar degeneration. [55]

We detected markedly divergent grey and white matter changes in *C9orf72*-positive and *C9orf72*-negative ALSFTD patients. In contrast to the widespread atrophy observed in C9 + ALSFTD, cortical thinning did not reach statistical significance in C9-ALSFTD. This is consistent with the more extensive cerebellar involvement associated with the *C9orf72* mutation [13, 19, 27, 28]. Cerebellar, cerebral and spinal changes have also been detected in presymptomatic GGGGCC hexanucleotide repeats expansion carriers [56–59]. It is noteworthy, however, that p62-immunoreactive TDP-43-negative neuronal cytoplasmic inclusions were noted in cerebellar granule cells irrespective of *C9orf72* status [15, 28]. Widespread cerebellar and cerebral degeneration have also been consistently noted in ALS and PLS cohorts without FTD [60–62]. Dysarthria, pseudobulbar affect, and cognitive deficits are commonly observed in ALS, and cerebellar pathology may contribute to these symptoms [63–67]. Interestingly, we detected higher cortical thickness in lobules I–II in *C9orf72*-negative ALSFTD compared to controls, which may be in line with the proposed compensatory role of the cerebellum in ALS [68–70].

Our findings may have clinical implications. Patients with clinical and genetic FTD subtypes attend a broad range of specialist including neurologist, psychiatrists and medicine for the elderly physicians. Clinical assessments may be heavily weighted towards cognitive and behavioural testing. If a cerebellar exam is performed at all, there is likely to be a greater emphasis on eliciting physical clinical signs. Post-mortem studies that confirmed cerebellar involvement in *C9orf72* highlighted the absence of overt ante mortem cerebellar signs such as ataxia without considering cognitive manifestations [27, 28]. It is conceivable that a formal cerebellar examination was not performed in some of these cases, and subtle cerebellar deficits may remain unrecognised. Since in our study lobule V degeneration was a consistent finding in all FTD subtypes, and this structure is a principal hub of cerebro-cerebellar sensorimotor networks, we suggest that formal cerebellar examination should be performed in all patients with suspected FTD. In addition, sequencing tasks (visual, verbal, behavioural and spatial) could be considered as a screening tool for cerebellum-associated cognitive dysfunction [6]. In those with apparent autosomal dominant inheritance who test negative for common FTD genes, it is important to consider SCA17; as it may initially resemble bvFTD [71]. The establishment of phenotype-specific imaging signatures and biomarker profiles may also aid the accurate categorisation of single subject datasets into relevant diagnostic, phenotypic or prognostic groups [72–76].

In addition to the lack of molecular profiling, a key limitation of our study is the sample size of our cohorts, particularly in those with PPA. Accordingly, our data need to be replicated in larger cohorts and validated by the dedicated assessment of the cerebellum post-mortem. Longitudinal radiological data acquisition may help to further elucidate the dynamic biological processes underpinning the progressive symptoms observed clinically [77]. Future cerebellar studies in FTD may benefit from complementing quantitative MRI analyses with FDG-PET to establish the comparative detection sensitivity of the two modalities. While previous PET studies captured cerebellar hypometabolism, no convergent patterns have been identified [16, 24, 25].

Our own findings, and the limited literature available, suggest that cerebellar degeneration is an important, albeit under investigated facet of FTD research, which merits dedicated clinical, imaging and post-mortem studies. The characterisation of cerebellar pathology in FTD is not merely an academic pursuit. The concomitant degeneration of interconnected infra- and supra-tentorial regions indicates connectivity-mediated propagation mechanisms, which may aid the identification of novel therapeutic targets. The demonstration of markedly divergent cerebellar signatures across the spectrum of FTDs serves as a reminder that FTD is a pathologically heterogeneous condition and the quest for ‘one drug for all’ is a naïve notion. In line with the principles of precision medicine, phenotype- and genotype-specific disease-modifying strategies are likely offer therapeutic benefits. Pioneering antisense oligonucleotide (ASO) studies in *C9orf72* give cause for optimism to target specific genotypes, and coordinated research efforts targeting tau may also pave the way to breakthrough individualised therapies. [57, 78] The refinement of clinical screening tools and the development of disease-specific imaging protocols may not only assist the accurate categorisation of suspected FTD patients, but serve as biomarkers in future clinical trials.

## Conclusions

Our data indicate unique cerebellar imaging signatures in FTD phenotypes with the selective involvement of specific lobules. It is conceivable that facets of behavioural and cognitive impairment previously exclusively attributed to supratentorial regions, may in part stem from cerebellar degeneration. Our findings highlight the involvement of infratentorial regions in FTD and support the evolving role of the cerebellum in cognitive and behavioural manifestations.
